# Application of asymmetrical configuration in electrochemical noise to investigate corrosion inhibition of aluminum alloy by *Ranunculus Arvensis*/*silver* nanoparticles

**DOI:** 10.1038/s41598-023-28443-0

**Published:** 2023-04-07

**Authors:** Ghazal Sadat Sajadi, Razieh Naghizade, Seyed Mohammad Ali Hosseini

**Affiliations:** Department of Chemistry, Shahid Bahonar University of Kerman, Kerman, 76175 Iran

**Keywords:** Corrosion, Electrochemistry

## Abstract

Asymmetric Configuration (As-Co) in the electrochemical noise (EN) was used to evaluate *Ranunculus Arvensis* / silver nanoparticles (RA/Ag NPs) as corrosion inhibitor for aluminum alloy (AA 2030) when gets exposed to 3.5% NaCl media. The ECN results of Asymmetric Configuration (As-Co) and Symmetric Configuration (Sy-Co) were interpreted using wavelet and statistical methods. The standard deviation of partial signal (SDPS) plots derived using of wavelet. The SDPS plot of As-Co demonstrated that the quantity of electric charge (Q) decreased with the addition of the inhibitor up to the optimum amount (200 ppm) due to the decreased corrosion rate. Moreover, the use of As- Co leads to record signal of one electrode and prevent recording extra signals arising from two identical electrodes which confirmed by the statistical parameters. The As-Co made of Al alloys were more satisfactory for estimating inhibiting effect of RA/Ag NPs compared to Sy-Co. Besides, aqueous extract of *Ranunculus Arvensis* (RA) plant as reducing agent mediates the synthesis of silver nanoparticles (RA/Ag NPs). The prepared NPs have been elaborately characterized using Field-Emission Scanning Electron Microscopy (FESEM), X-Ray Diffraction (XRD) analysis, and Fourier-Transform Infrared Spectroscopy (FT-IR) that revealed suitable synthesize of the RA/Ag NPs.

## Introduction

Corrosion is a normally occurring unconstrained peculiarity which prompts a change of metals or alloys in their steady types by responding with environment chemically or electrochemically^1^. Among metals and alloys the Aluminum alloys of the 2000 series such as Al-Cu-Mg-Pb (AA2030) alloy is widely used in various industries due to good corrosion resistance and etc.^2^. The main corrosive agents of constructions are Cl^-^, H_2_O, SO_4_^2-^, H_2_S, CO_2_, and dissolved O_2_^3–5^. To protect metals surface from corrosion in these aggressive media, consumption inhibitors are added. The corrosion inhibitors are organic or inorganic builds which are utilized in modest quantity to impede the corrosion rate significantly^6,7^. At the point when inhibitor is introduced in to the aggressive media, safeguards the metals surface from dissolving by adsorbing on the outer layer of metals in this way making a boundary for mass and charge move. Because of the expanded worry of climate controlling organizations of different nations, the utilization and production of inorganic corrosion inhibitors are kept away, leading to promote research focus on look for the new and earth harmless corrosion inhibitors called green corrosion inhibitors for the most part^8^.

The greater part of exploration over nanotechnology includes the synthesis and stabilization of a scope of nanoparticles by physical and composed strategies, which are not environmentally sound. Therefore, there is presently a rising need to introduce a harmless matter including non-poisonous materials which don't contain heavy metals. Because of minor size, mechanical, electromagnetic, optical and biochemical properties of nanoparticles, they can be utilized to retard corrosion of the metals and alloys. Among the metallic particles, considerable attention has been paid to silver nanoparticles (Ag NPs) due to their wide applications in Nano medicine, electronics, catalysis, and optics regions^9–12^.

Electrochemical noise (EN) estimating the variation of current and potential in the electrochemical framework during corrosion activity has been effectively used to concentrate on the localized corrosion of Al alloy^13–15^. The EN measurement was conducted under the open circuit potential (OCP) with a cell having two identical working electrodes (WEs) connected with a zero-resistance ammeter (ZRA)^16^. Recently confirmations have uncovered that asymmetrical configuration (AS-Co) having two electrodes with various sizes promotes noise identification^17–20^. According to Shahidi et al.^17^, using an asymmetric system with a difference in size between the two WEs lets to the recording ECN of one electrode while permitting to continue natural spontaneous corrosion. In addition, advantages of utilizing asymmetric electrodes prepared of AA6061 and AA2024 alloys in chloride media at varies pH values revealed by Bahrami et al.^18,19^. As Sajadi et al.^20^ indicated, asymmetrical electrodes made of copper allow to study what is happening on one of the two electrodes an enhance the detection of pitting corrosion.

The EN analysis is performed using two main approaches, including statistical and spectral techniques, which require pretreatment prior to analyzing the data due to DC drift and nonstationary status. Wavelet transformation (WT) is an adapted signal processing method to analyze EN transients that do not require stationary or linear conditions while showing a strong potential for simultaneously differentiating time and frequency domains^21–26^. The graph of the standard deviation of partial signal (SDPS) versus crystal is one of the ways to show the results acquired from the wavelet investigation process. The corrosion timescale and frequency range alongside the relationship with the number of crystals are shown in Table 1. In order to, as the number of crystals expands, the timescale and frequency range of corrosion additionally increase^17,18,27^. A few boundaries like standard deviation, skewness, and kurtosis can be processed by statistical investigations. The first one demonstrates the electrochemical exercises on the outer layer of sample. The subsequent one estimates balance about the average, and the latter shows the overhead or flat distribution^28–31^.

**Table 1:** Frequency along with timescale ranges in sampling frequency 4 Hz.

Crystal name	Frequency range/Hz	Timescale range/s
d1	4–2	0.25–0.5
d2	2–1	0.5–1
d3	1–0.5	1–2
d4	0.5–0.25	2–4
d5	0.25–0.125	4–8
d6	0.125–0.0625	8–16
d7	0.0625–0.0312	16–32
d8	0.0312–0.0156	32–64
d9	0.0156–0.0078	64–128

The main aim of present study is to employ As-Co made of Al alloy in EN method to improve the measuring of the corrosion inhibitory effect with different concentration of the inhibitor. Furthermore, novel green synthesis of Ag NPs is done by RA extract. The RA/Ag NPs were characterized using Field-Emission Scanning Electron Microscopy (FESEM), X-Ray Diffraction (XRD) analysis and fourier-transform infrared spectroscopy (FT-IR). The inhibitory ability of the prepared NPs against Al alloy corrosion in 3.5% NaCl solution is systematically investigated employing ECN and electrochemical impedance spectroscopy (EIS) techniques. The EN data were analyzed by SDPS plots and statistical parameters. The corrosion morphologies were observed by optical microscopy.

## Materials and Methodology

### Materials

All materials that utilized in this research for preparation of the electrolytes, *Ranunculus Arvensis* extract and RA/Ag NPs are 96% Ethanol, 99% Silver Nitrate, 99% Chloride Sodium and 96% Acetone.

### Preparation of RA extract

The fresh RA plant were purchased from the local markets in the Iran -Kerman, to extricate the RA, the samples rinsed under running water and dried in a light-proof area. Total phenolic concentrations are higher in the shade drying that gave the greatest volume of antioxidants^32^. Taking effective method is concerned, we have picked a shade drying in view of this investigation. Subsequent to drying, the leaves were transformed into powder with 2–10 mm particles. Following, the suspension of 50 g of powder was placed into 400 mL analytical reagent grade ethanol at temperature of 25 ± 2 °C for a time of ideal time period i.e. 15 days. A while later, the suspension was separated by Whatman No.1 filtration paper, and then to evaporate the ethanol exposed to Heating Mantle (MS-E105). Finally, the resultant 120 mL concentrated RA extricate (RA separated and the remainder of ethanol with a proportion of 1:2 individually) was utilized for additional study.

#### Declaration for the usage of plant materials

We declare that in this research, we did not use or not going to use any plants (either cultivated or wild) irrespective of any location. Experimental research and field study in this study has complied with the IUCN Policy Statement on Research Involving Species at Risk of Extinction. The use of plants in the present study complies with international, national and/or institutional guidelines.

### Preparation of RA/Ag NPs

Silver particles (Ag^+^) were derived from silver nitrate (AgNO_3_) to incorporate RA/Ag NPs. Around 800 mL of 0.1 M AgNO_3_ added to 120 mL of the reduced RA extract in an Erlenmeyer jar at 25 ± 2 °C. The color of aqueous medium was changed quickly from dull green to yellowish-green flagging the arrangement of AgNPs^33–35^. Then, the fluid media were permitted rest for to 3 days at room temperature. The RA/Ag NPs were extricated by centrifuging at 5000 rpm for 20 min and afterward permitted the reagents to dry. The green strong deposits of RA/Ag NPs were washed three times by double distilled water which was finally evaporated. The prepared RA/Ag NPs powder, were utilized as green inhibitor.

### Preparation of solutions

The corrosive media 3.5% NaCl were prepared by diluting analytical grade Merck NaCl with double distilled water, which introduced blank (without inhibitor). Before each analysis, the test media were prepared freshly by blending the inhibitor with the corrosive media, and then sonicated. Tests were directed two times to confirm repeatability.

### Preparation of the Al electrodes for electrochemical test

The samples for the corrosion testing were made of AA 2030 Al alloy. Table 2 shows the chemical composition of the alloy. Preparation of two configuration of electrochemical cell took place, including symmetric configuration (100–100 mm^2^) and asymmetric configuration with a large difference in size of two WEs (3–300 mm^2^) denoted as Sy-Co and AS-Co, respectively. Therefore, the only difference between two the configurations is the area of the electrodes. Connecting of the electrodes to a wire made of copper were done then they were totally covered with glue such that one surface, with the defined region, presented as the WE surface. Silicon carbide papers (at a scope of 200#–3000#) were utilized to abrade and polish the alloy, following distilled water utilized for cleaning, and Acetone was used to degrease before each measurement.

**Table 2:** Chemical composition of Al alloy (wt%).

Element	Si	Fe	Cu	Mn	Mg	Bi	Pb	Zn
wt%	0.8	0.7	3.9	0.5	0.87	0.19	1.2	0.37

### Electrochemical analyses

Two face-to-face Al electrodes with a gap of 2 cm were situated in the media to frame the electrochemical configuration. Measuring of ECN signals were done in 900 s After 30 min from the immersion time in 3.5% NaCl solution at the absence and presence of five different inhibitor concentrations (50–250 ppm). Utilization of the orthogonal Daubechies wavelets of the fourth order (db4) assisted with playing out wavelet procedure and the frequency of 4 Hz was utilized for the sampling frequency of the ECN information. Estimation of statistical parameters is completed pursuing the wavelet transform technique. Three-electrode system was used to perform the EIS tests on Al alloy as WE, platinum as counter electrode, and Ag/AgCl as reference electrode.

At open-circuit potential in the frequency range 100 kHz–10 mHz, applying a 10 mV sinusoidal perturbation was considered for the EIS measurements. Calculations of the inhibition efficiency (IE) were estimated by polarization resistance in absence (R_p,blk_) and presence (R_p,inh_) of inhibitor in the following equation^36^:

1 $$\%IE=\left(\frac{{R}_{p. inh}-{R}_{p. blk}}{{R}_{p. inh}}\right)\times 100$$

The Nyquist and Bode curves were derived by EIS in a 3.5% NaCl electrolyte with and without different inhibitor concentrations of 50–250 ppm. Matlab software was used for processing ECN data and Nova software was used for analyzing EIS curves.

### Characterization

ield-Emission Scanning Electron Microscopy (FESEM EM-8000) was applied to obtain the structures of the RA/Ag NPs. XRD spectra were performed on a Shimadzu XRD-6000 diffractometer(Japan) using Cu kα radiation, and Fourier transform infrared (FT-IR) spectra were recorded on a Nicolet 10 transform infrared spectrometer in the region of 525 cm^−1^ to 4000 cm^−1^. To examine the NPs inhibitor performance, electrochemical research such as ECN and EIS were measured by the Auto-Lab device (302 N potentiostat, Netherlands). The investigations of alloy surface morphology, submerged in corrosive media in the presence and absence of the optimal concentration of the RA/Ag NPs, were done by optical microscopy (Leica zoom 2000 model). For the last test, WEs were precisely polished, and submerged in 3.5% NaCl media in the both manners (with and without) of the inhibitor for around 24 h at room temperature and afterward eliminated and dried.

## Results and discussion

### Characterization of the NPs

**Figure 1:**
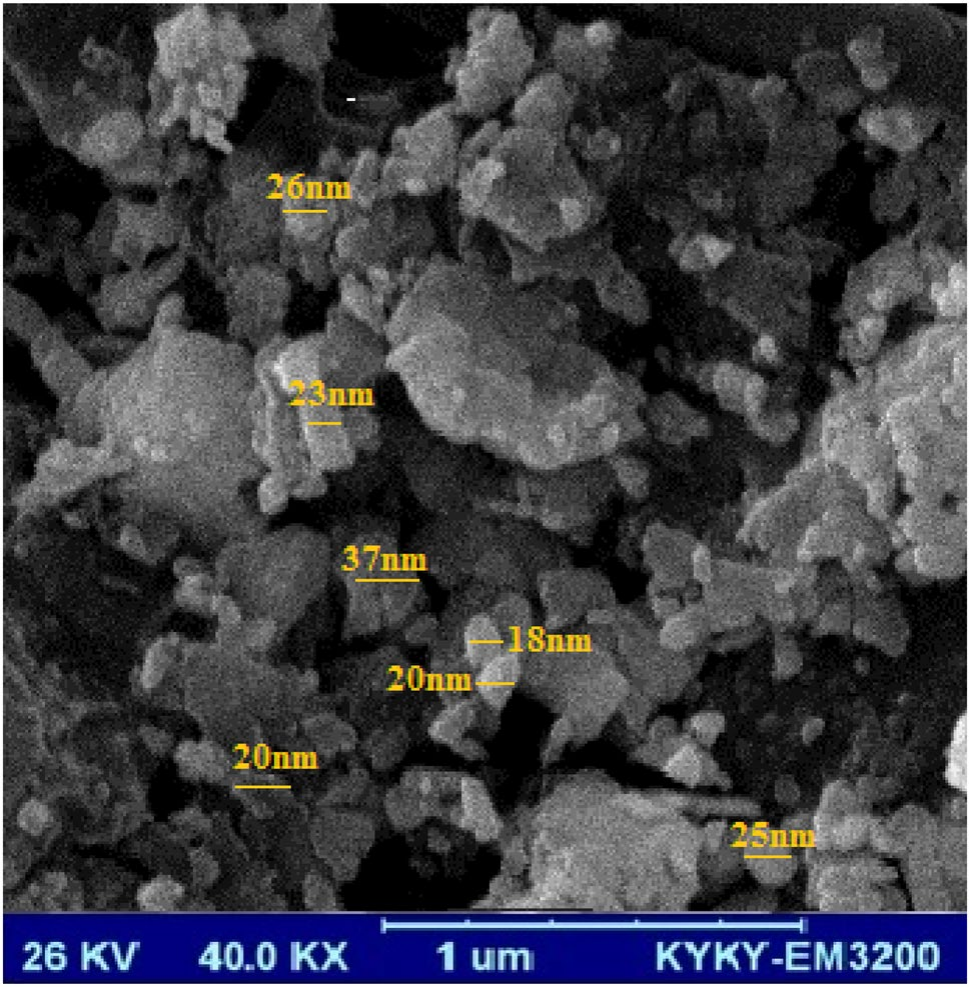
SEM images of RA/Ag NPs.

FESEM initially checked the synthesized NPs’ morphology to highlight the possibility of the formation of particulate materials prior to more characterizations. The FESEM micrograph, shown in Fig. 1, reveals distinguished crystalline polygonal shape of RA/Ag NPs with a low amount aggregates or agglomeration formation. Approximately crystalline with the particles size differing from 18 to 37 nm and the average size of the particles estimated 24.0 ± 1.9 nm validating suitable synthesize of the NPs. Since this synthesis procedure is safe, easy, rapid, inexpensive and eco-friendly method which does not require high pressure, energy, temperature and toxic chemicals for production. Therefore, the green synthesize of the RA/Ag NPs are more appropriate than those one obtained by chemical reagents^37–39^.

**Figure 2:**
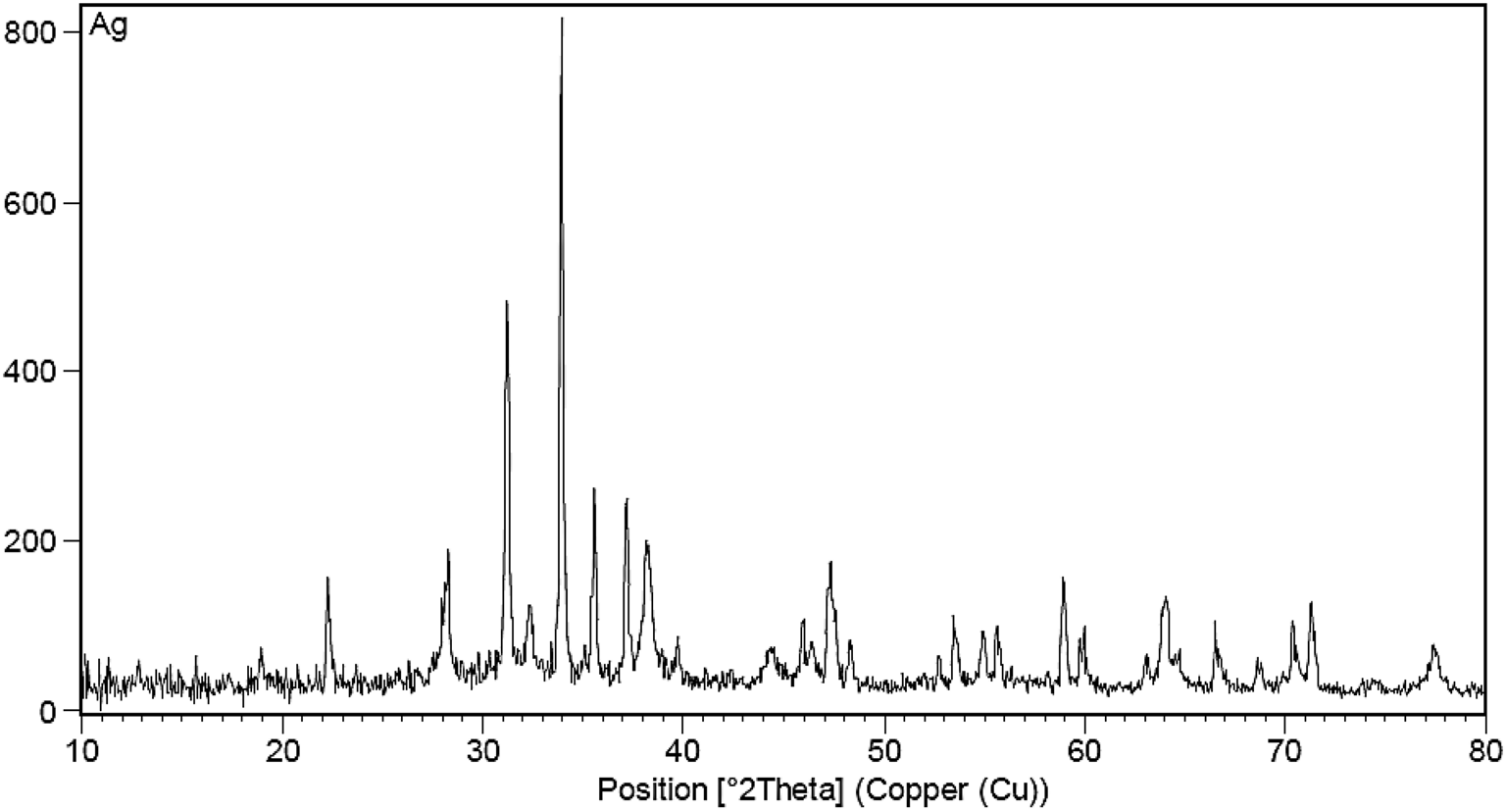
XRD analysis of prepared RA/Ag NPs.

In addition, the NPs’ XRD pattern was prepared and shown in Fig. 2, indicating the peaks at 2θ values 22.28°, 28.23°, 31.21°, 33.92°, 37.17°, 38.22° and 47.28°, corresponding to the reflections of (Peak List). subsequently, there is good accordance with the standard diffraction data of Ag NPs (JCPDS card no: 00-001-0961 and 01-087-0717).

Plants for the synthesis of NPs have wide variety of active agents that can aid in bio-reduction of Ag + ions, subsequently forming the Ag NPs. Leaves, stems, barks, flowers, seeds, and their derivatives have all been successfully used for the biosynthesis of nanoparticles^40,41^. The previous article reported the active agents found in RA extract^42^, which allow stabilization and reduction of Ag^+^ ions in this study.

**Figure 3:**
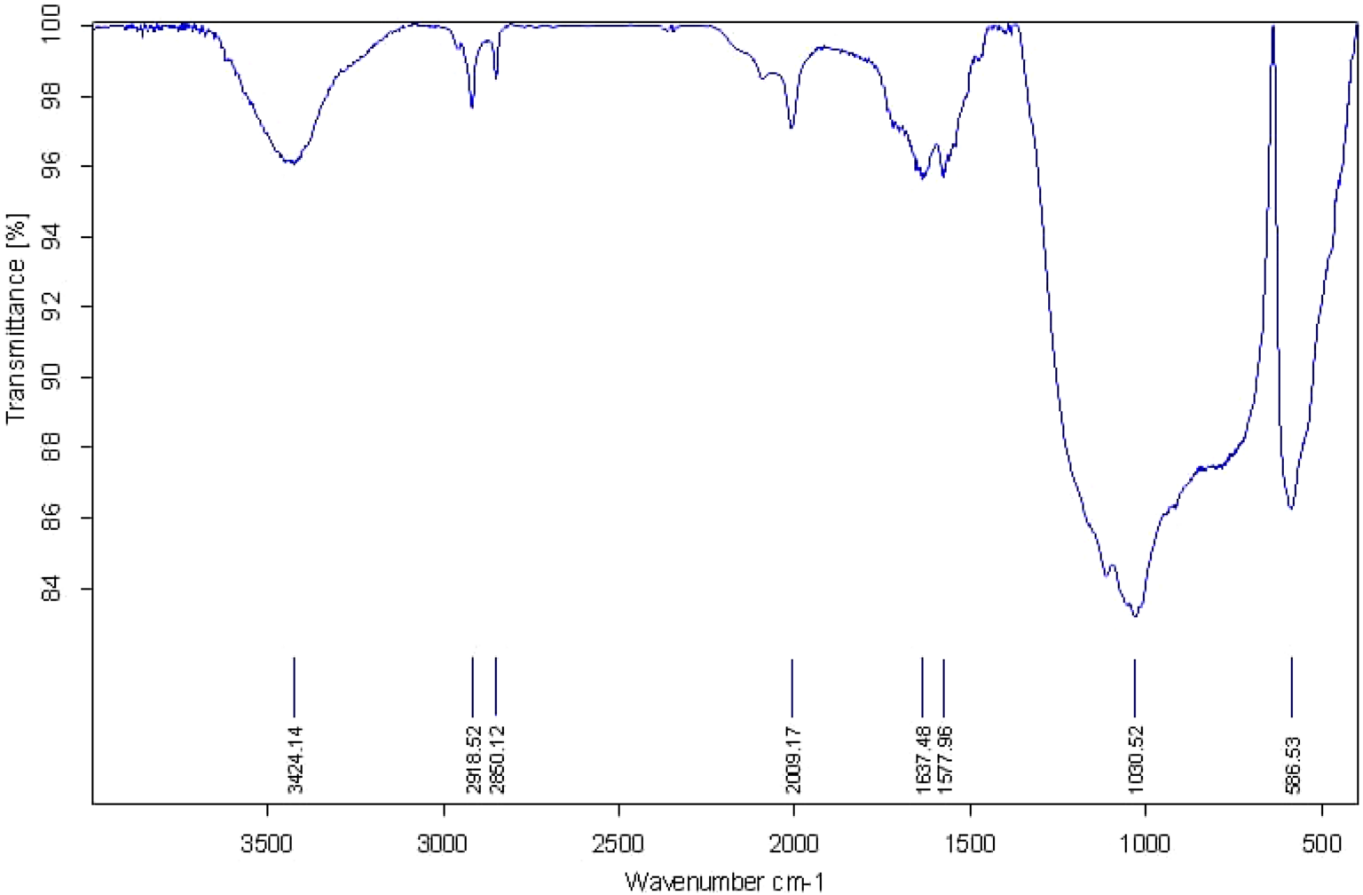
FTIR spectra of RA/Ag NPs prepared using *Ranunculus Arvensis* extract.

Figure 3 illustrates the FTIR used to identify several active functional groups in the RA/Ag NPs. The peak is shown at 1030 cm^−1^ by the NPs’ spectrum, highlighting the C–O (alcohol, carboxylic acid, ester and ether) groups stretching. The other peak at 3424 cm^−1^ is associated to the stretching of O–H (alcohol, phenolics, flavonoids, and so on), and at 1577 cm^−1^ correspond to C=C (aromatic). The peaks at 2918 and 2850 cm^−1^ correspond to C–H stretch of methylene groups, revealing higher prominence in the Ag NPs spectrum. Besides, the sharp peak at 586 cm^–1^ may be associated with Ag specific functional groups in the fingerprint region, explicating the great stability of RA/Ag NPs green synthesis^43^.

### EN measurements

The SDPS and statistical methodes are the main analyses employed to evalute the EN signals. The trends of direct current were eliminated by using the WT procedure prior to the EN analyses. The WT represents the EN signal at varias timescales as crystal numbers. The frequency range of each crystal is obtained by the following equation:

2 $${(f}_{1}\cdot {f}_{2})= {2}^{-j}{f}_{s}\cdot {2}^{1-j}{f}_{s}$$

where f_s_ is sampling frequency, and j is the number of the crystal. The scale range of each crystal is given by the below equation^27^:

3 $${(I}_{1}\cdot {I}_{2})={2}^{j}\Delta t\cdot {2}^{1-j}\mathrm{\Delta t}$$

where Δt is the sampling interval (Δt = 1/f_s_). Table 1 shows the frequency and scale range of the case in which J = 9 and f_s_ = 4 Hz. The inverse wavelet transform can produce partial signals of the original signal. Each partial signal is a signal which resembles the fluctuations of the original signal at a particular timescale. The standard deviation of partial signal (SDPS) can indicate the variations in the intensity of the partial signal about its mean, which could be an indication of the intensity of electrochemical activity on the surface of the electrodes within a particular interval of frequency^27^.

#### Asymmetric configuration (As-Co)

##### SDPS analysis

**Figure 4:**
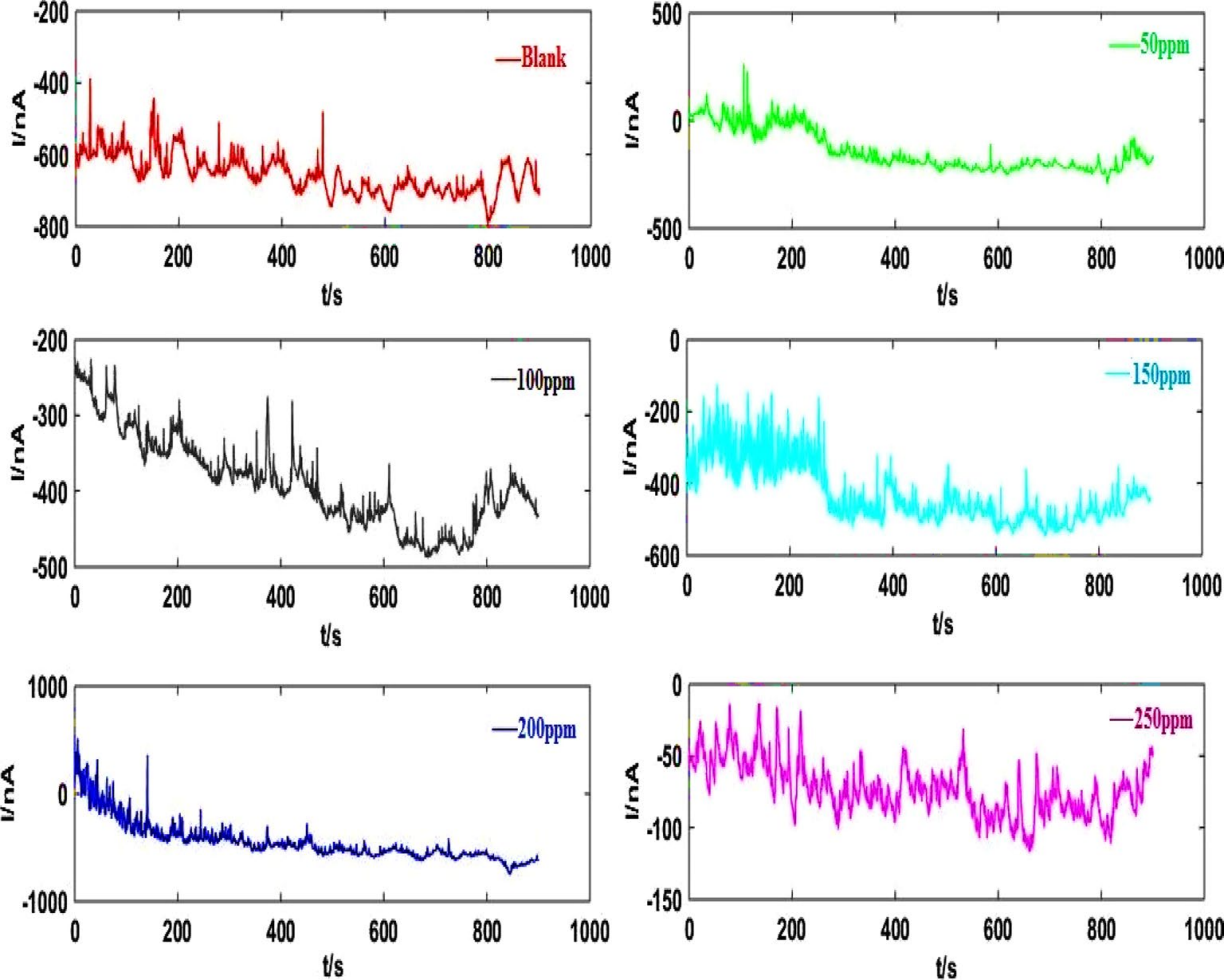
EN current records before elimination trend of As-Co in absent and presence of varies concentrations of RA/Ag NPs.

**Figure 5:**
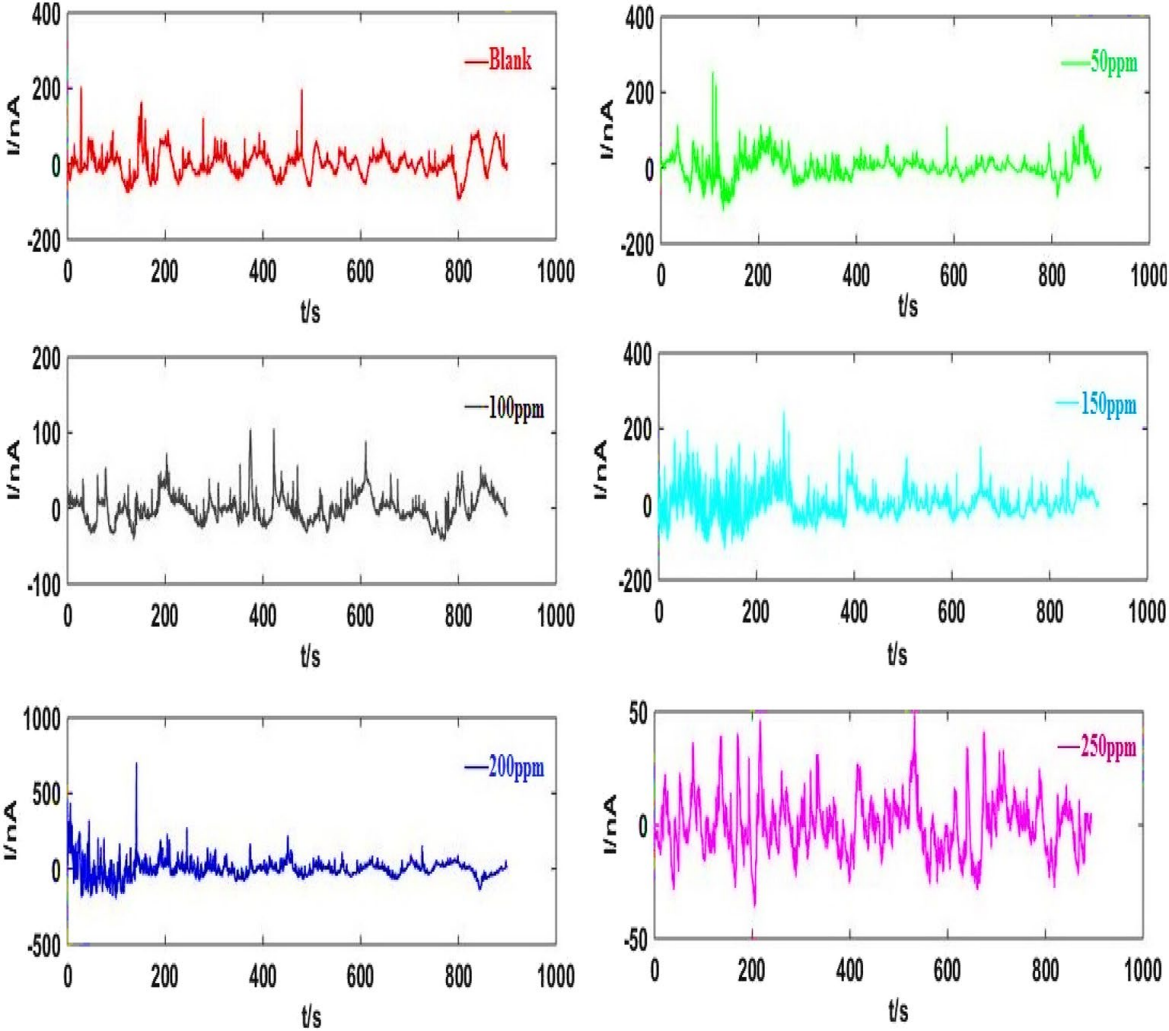
EN current records illustrated in Fig. 4, after elimination trend.

Measurement of the EN data that corresponded to nominally As-Co was conducted when various RA/Ag NPs concentrations after 30-min immersion time (Fig. 4). As indicated in Fig. 5, the ECN signals arising from asymmetric electrodes are unidirectional and they are without any changes in the practical data after removal of the current trend using wavelet analyses. Overall, two major EN signals are considered, including the signals that present distinctive-shaped transients and the ones created by irregular fluctuations^44^. The first type includes signals provided by As-Co and is usually regarded as those containing more information compared to others. The EN transients’ timescale determines the number of required crystals.

**Figure 6:**
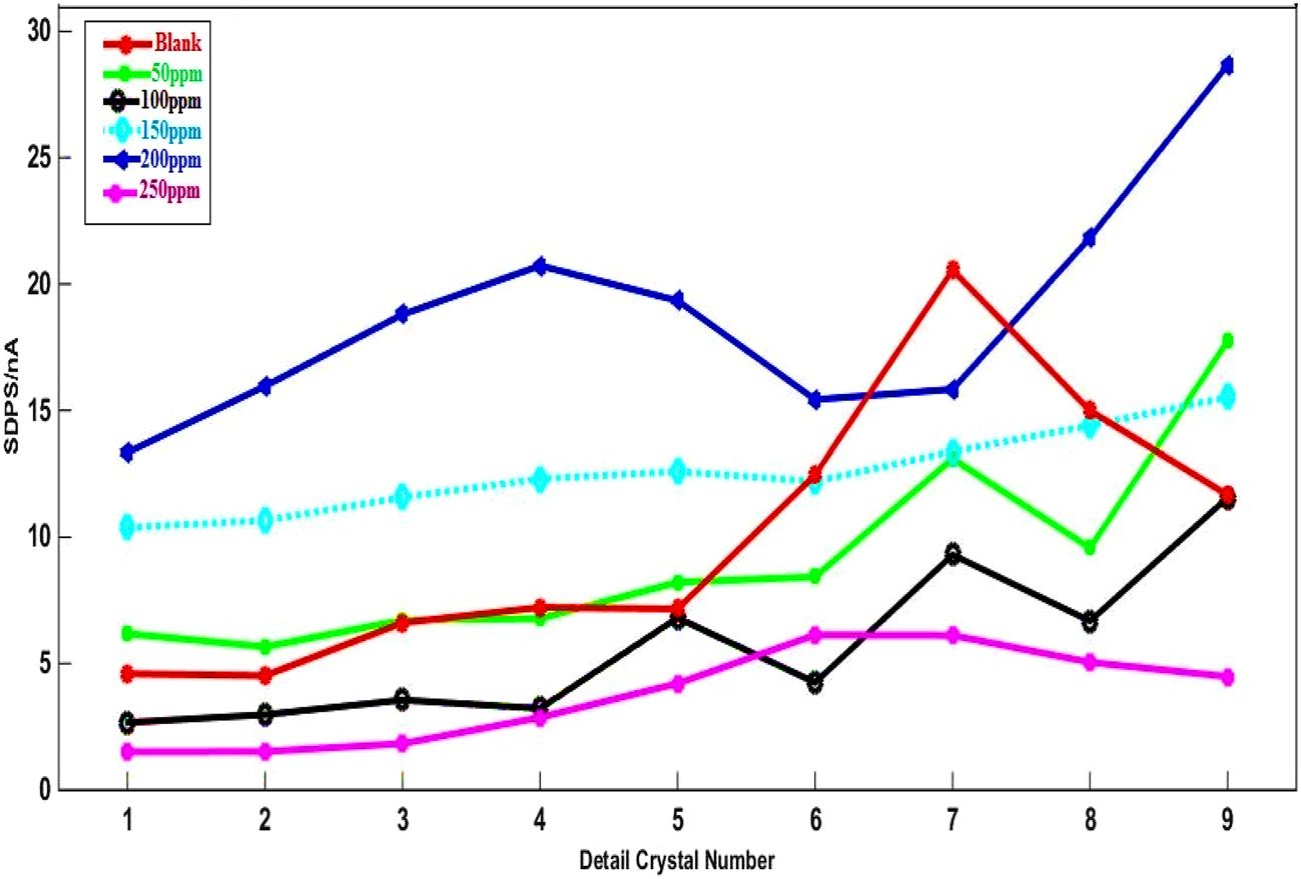
SDPS plots of As-Co in absent and presence of varies concentrations of RA/Ag NPs.

Signal blank includes longer timescales and SDPS values of the EN signals because of the greater corrosion severity when the inhibitor is absent. As Fig. 6 shows, a nearly 20-s time period is taken by the transient in signal blank, corresponding to the d7 crystal (Table 1). The employment of WT aimed at decomposing single sets of data points and obtaining SDPS plots of signals represented in Fig. 6. To study the localized corrosion a coulombs counting method, known as Coul Count, was already introduced^44,45^. The equation below is used to calculate the total amount of noise charges Q(t) at the time t, exchanged between two WEs^45^:

4 $$Q\left(t\right)=\sum_{i=0}^{t/\Delta t}|{I}_{i}|\Delta t$$

Averaging absolute current noise data, |Ii| (e.g. 600 data points for 1 min) and corresponding adjustment of Δt (1 min) leads to data reduction. Calculation of the total noise charge amount can be performed more conveniently using the partial signals taken from EN current data by wavelet analyses rather than the hardware filtered noise signal (with fluctuations > 0.1 Hz). According to Section "Introduction", every partial signal has similarities to the original signal fluctuations at a specific frequency interval. Thus, using the SDPS plots can be suitable to calculate the noise charge amount at a specific frequency interval. The predominant transients are associated with metastable pits, whose development leads to the flow of a quantity of electric charge in the circuit, calculated using the below equation^46^:

5 $$\left( {Q = i_{max} \times \tau_{max} } \right)$$

In which i_max_ shows the SDPS value at the maximum peak crystal (d_max_) and τ _max_ represents the average time width of d_max_ crystal. The effectiveness of the corrosion inhibition can be defined using the following relation^46^:

6 $${IE}_{EN}\left(\%\right)=\frac{Q-{Q}^{^{\prime}}}{Q}\times 100$$

In which, Q and Q^'^ represent the noise charges in the uninhibited and inhibited cases, respectively. As shown by the blank signal SDPS plots in Fig. 6, d7 crystal has the highest SDPS value of 20 nA with an average time width of 24 s. Table 3 also summarizes the parameter values taken based on the SDPS plots of Fig. 6, including Q and IE. As indicated, when NPs are added to the NaCl solution, the values of noise charges reduced. The minimum value of the Q is recorded at NPs’ inhibitor concentrations of 200 ppm, leading to improve the efficiency of the inhibition, with the highest value reported equal to 87.50%. Which indicate more inhibitor is absorbed to protect the Al alloy’s surface. Greater amounts of Q arise blank sample than other instances, resulting in higher rates of corrosion^47^.

**Figure 7:**
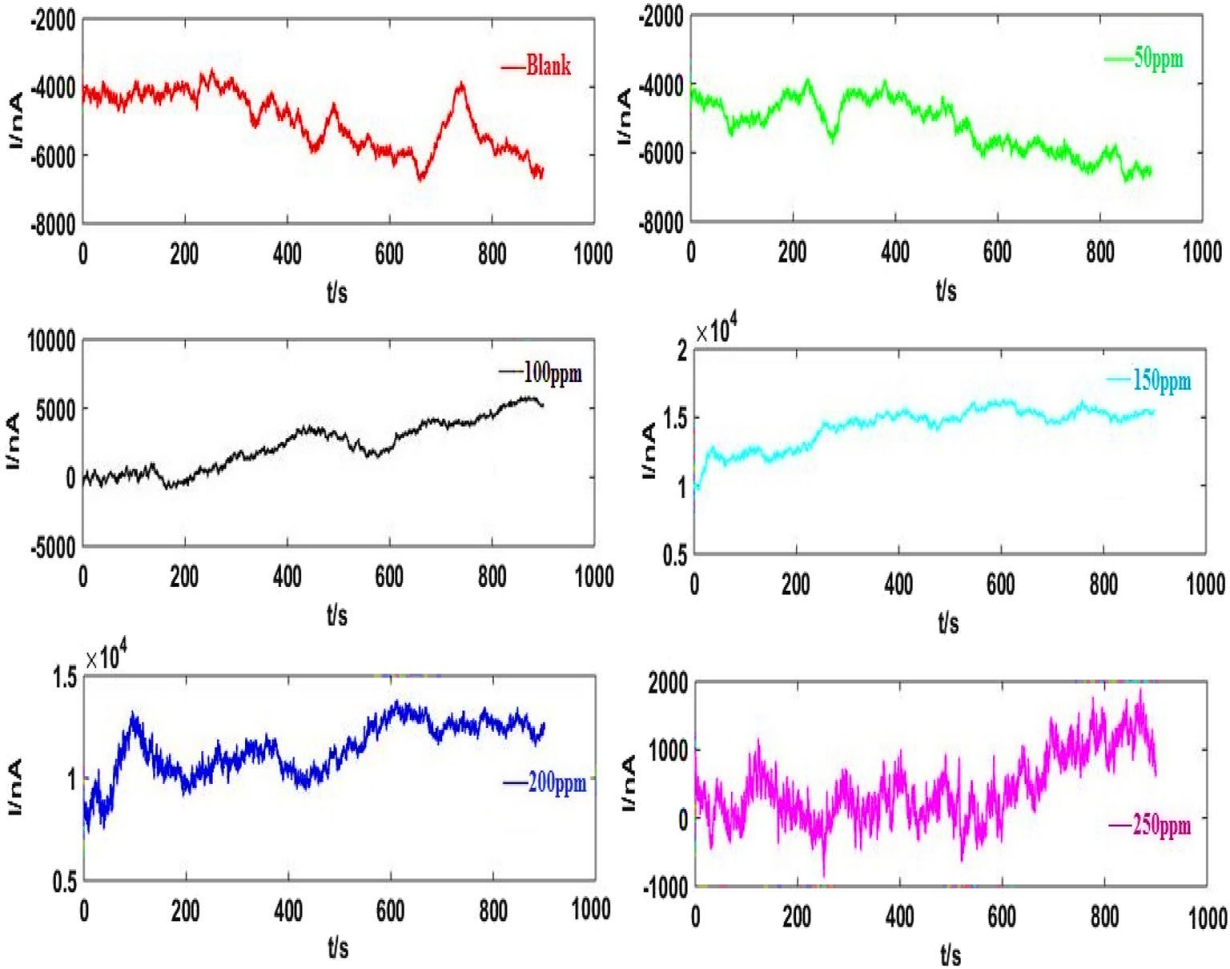
EN current records before elimination trend of Sy-Co in absent and presence of varies concentrations of RA/Ag NPs.

**Table 3:** The time scales of the predominant transients of SDPS plots related to As-Co and Sy-Co made of Al alloy in 3.5% NaCl.

Configuration	C/ppm	SDPS_max_/nA	d_max_(obv)	d_max_(real)	τ_max_/S	Q/nCoul	IE%
Asymmetric	Blank	20	d7	d7	24	480	–
	50	12	d7	d7	24	288	40.00
	100	9.2	d7	d7	24	220	54.16
	150	12.5	d5	d5	6	75	84.37
	200	20	d4	d4	3	60	87.50
	250	5.8	d6	d6	12	69.6	85.50
Symmetric	Blank	300	d9	d7	24	7200	–
	50	95	d7	d7	24	2280	68.33
	100	90	d6	d7	24	2160	70
	150	150	d7	d5	6	900	87.50
	200	200	d7	d4	3	600	91.66
	250	105	d8	d6	12	1260	82.50

##### Statistical analysis

Table 4 also shows the results of the ECN signals statistical parameters taken from Fig. 5. It is noteworthy that the signal amplitude and number affect the standard deviation (SD)^48^. As shown in Table 4, the ECN signals’ SD are higher in the blank solutions compared to those obtained from the other inhibitor concentrations. The lower SD value is related to current signals of the inhibitor’s 200 ppm concentration. Such SD differences may be associated with the lower signal amplitude and numbers of signals in the presence of the inhibitors due to their greater kurtosis compared to the blank signals (Table 4). Moreover, the higher values of kurtosis result from lower number of signals, confirming the above argument^48^. The higher skewness is supposed due to uni-direction current signals of the inhibitor’s 200 ppm concentration (Table 4). The anodic and cathodic reactions occurring on the small and large WE, respectively, lead to this uni-directionality. Because of the higher thickness of protective film on the WE when optimum concentrations are present, inhibiting cathodic reactions (particularly on the small one) due to less space to carry out reduction reactions. Consequently, the signals that arises from the 200 ppm concentration of the inhibitor show greater uni-directionality compared to the other concentrations and the case of blank solutions (Fig. 5) as stated above given the insufficient space for reduction in the small WE^21,49,50^.

**Table 4:** Statistical parameters obtained of current signals arising As-Co and Sy-Co made of Al alloy in absent and presence of varies concentrations of RA/AgNPs.

Configuration	C/ppm	SD	Skew	Kurt
Asymmetric	Blank	33.58	0.47	1.69
	50	29.80	0.49	3.60
	100	19.16	0.87	2.13
	150	18.92	0.95	2.96
	200	11.91	1.41	10.77
	250	12.47	0.91	8.47
Symmetric	Blank	392.30	0.04	0.88
	50	292.47	-0.03	0.47
	100	379.52	-0.02	0.55
	150	337.14	-0.02	0.05
	200	245.23	0.07	1.19
	250	265.11	0.01	0.11

#### Symmetric configuration (Sy-Co)

##### SDPS analysis

**Figure 8:**
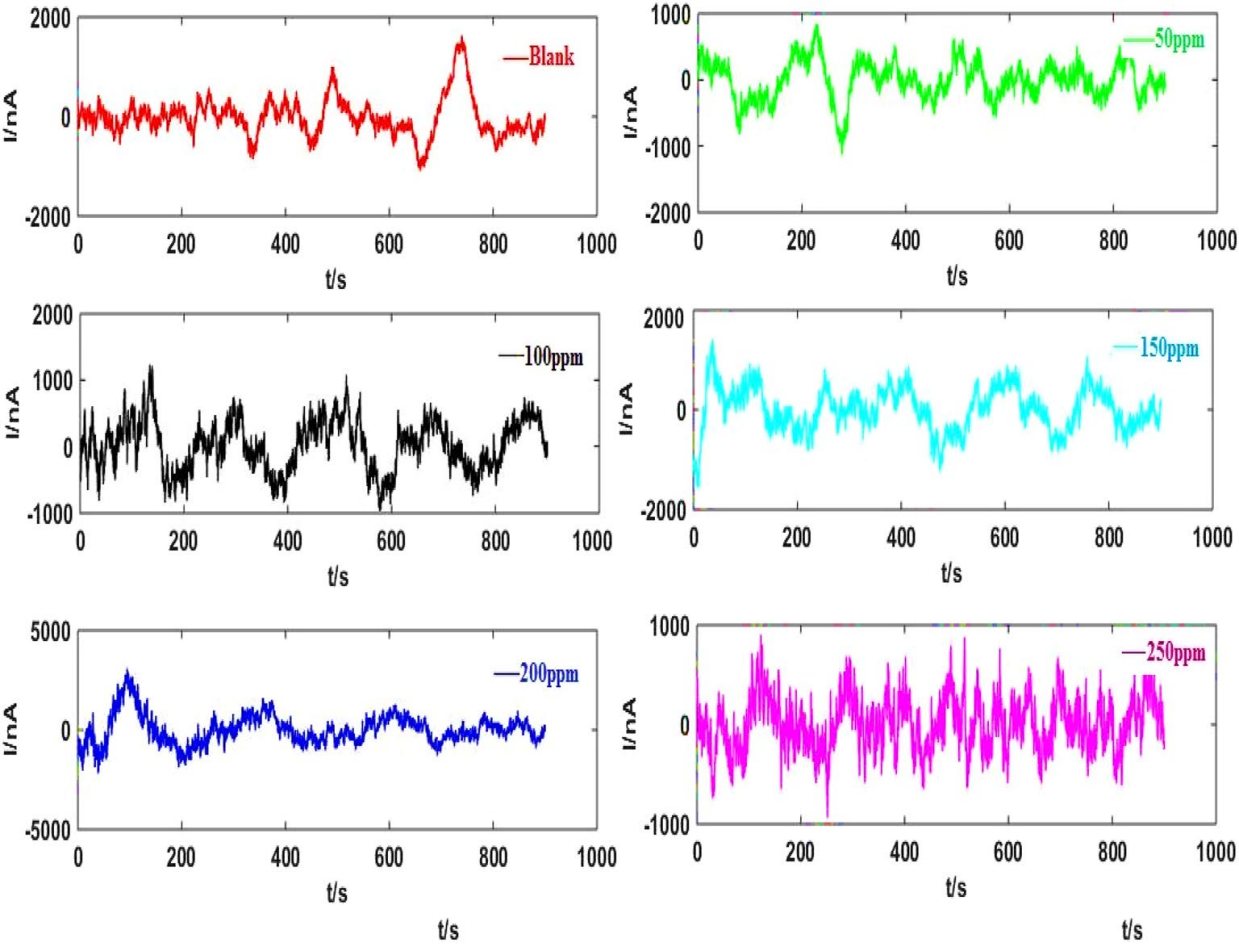
EN current records illustrated in Fig. 7, after elimination trend.

**Figure 9:**
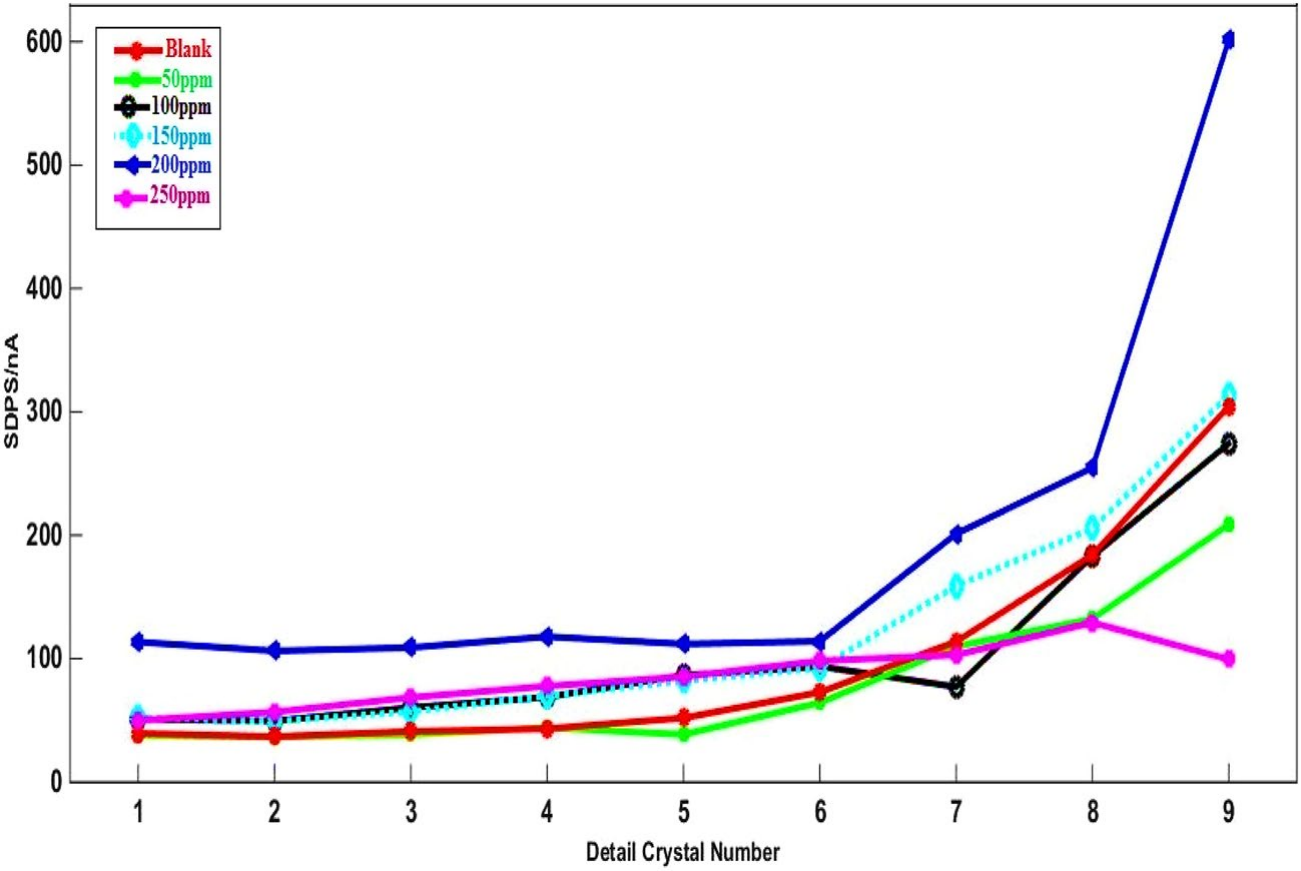
SDPS plots of Sy-Co in absent and presence of varies concentrations of RA/Ag NPs.

**Figure 10:**
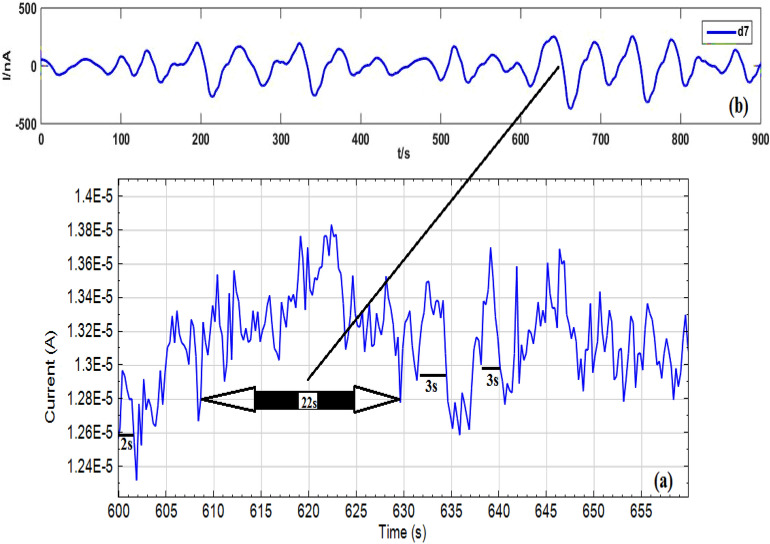
(**a**) The original signal of Sy-Co of RA/AgNPs 200 ppm concentration (**b**) the d7 partial signal.

Figure 8 indicates the bidirectional EN signals following the wavelet trend removal of original signals (Fig. 7), which achieved from Sy-Co made of Al alloys using a 3.5% NaCl solution when various RA/Ag NPs concentrations were absent and present. Figure 9 shows the SDPS plots, taken from the EN signals after trend removal by WT. According to Fig. 9, no evident maximum pick locations are exhibited at some inhibitor concentrations, necessitating the comparison of the partial and original signals to find out the predominant transients’ scale^17^. As an instance, comparison of the two types of signals associated with 200 ppm was carried out to ensure the d7 crystal (transients with 16–32 s time scale according to Table 1) accuracy. In Fig. 10a,b show several peaks for the d7 crystal partial signal under different times (including 610 s). Values of < 16 s are reported for the two transients at 610 s. Yet, detailed investigations show their combination of several overlapping instead of single transients. Thus, the selection of J = 7 as scale range to examine the transients is not acceptable because all single transients of the EN signals had a duration of < 4 s. It can be concluded that although the maximum peak of the SDPS plot of Sy-Co signal at inhibitor concentrations of 200 ppm is at the position of d7 crystal, the actual time scale of this signal corresponds to d4 crystal. The comparison of the partial and original signals will not be required in the As-Co observable maximum peak location, while the case of Sy-Co should undergo such comparisons^20^. Table 3 presents the actual and apparent time scales related to transients taken from Sy-Co.

##### Statistical analysis

Table 4 lists the statistical parameters following the Sy-Co signals trend removal under various concentrations, indicating higher SD values for the Sy-Co electrodes’ noise signals compared to those obtained for As-Co. Such differences in SDs could be associated with the greater number (not amplitude) of signals taken from Sy-Co due to their lower kurtosis compared to As-Co signals with larger signal amplitudes^17^. It is noteworthy that As-Co is used to prevent overlapping of the signals while improving the detection of the noise through enhanced amplitude^51^. Therefore, As-Co can be suitable candidates for the investigation of the corrosion inhibition characteristics due to their fewer noises. As shown in Table 4, bidirectional signals, including the case of Sy-Co, lead to zero skewness (Fig. 8). It can be due to the anodic and cathodic reactions on both identical work electrodes under similar reaction rates. Resulting only half of the generated electron of anodic site flows through ZRA because of the produced electrons division between the cathodic sites of two electrodes. The electron transfer takes place to the electrode which has the higher reduction reaction rate in the case of any differences between the reduction reaction on the WEs^20^.

### EIS measurements

**Figure 11:**
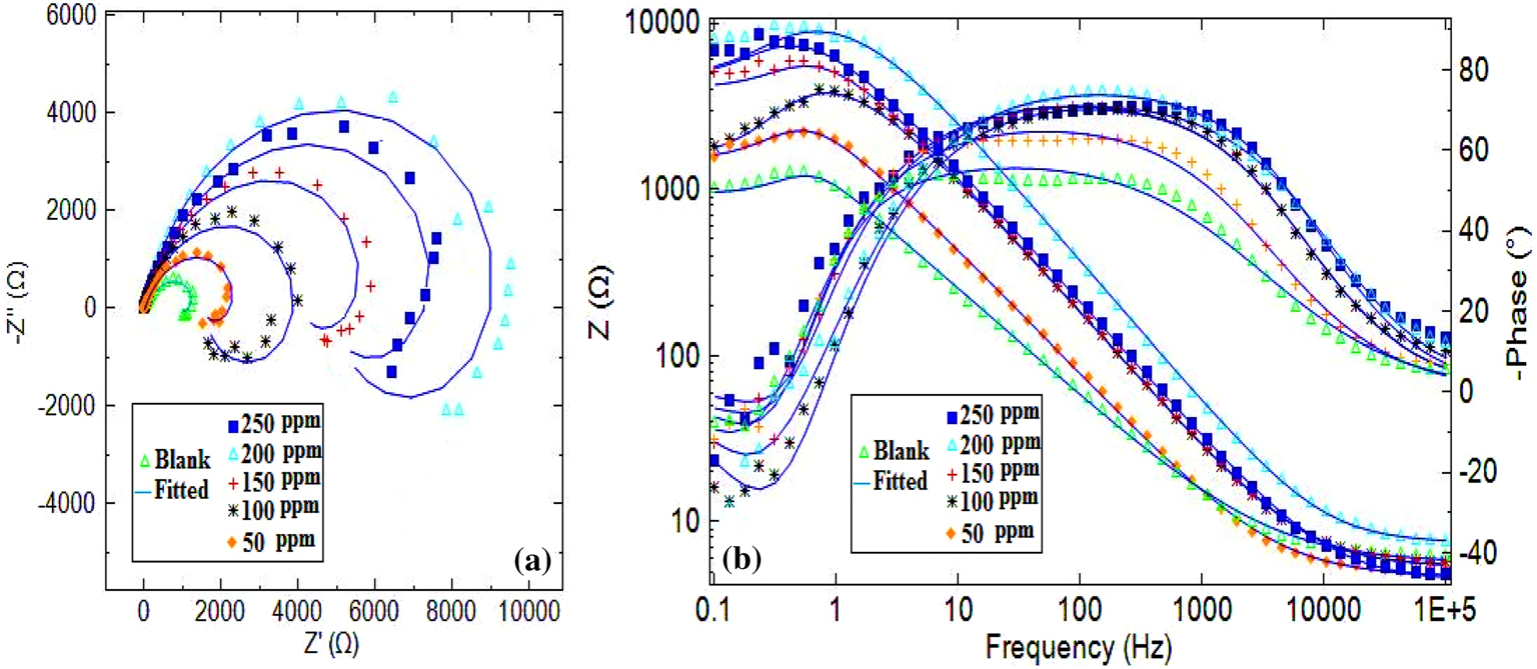
(**a**) The Nyquist and (**b**) Bode diagrams for Al alloy in corrosive media in the absent and present of RA/AgNPs.

**Figure 12:**
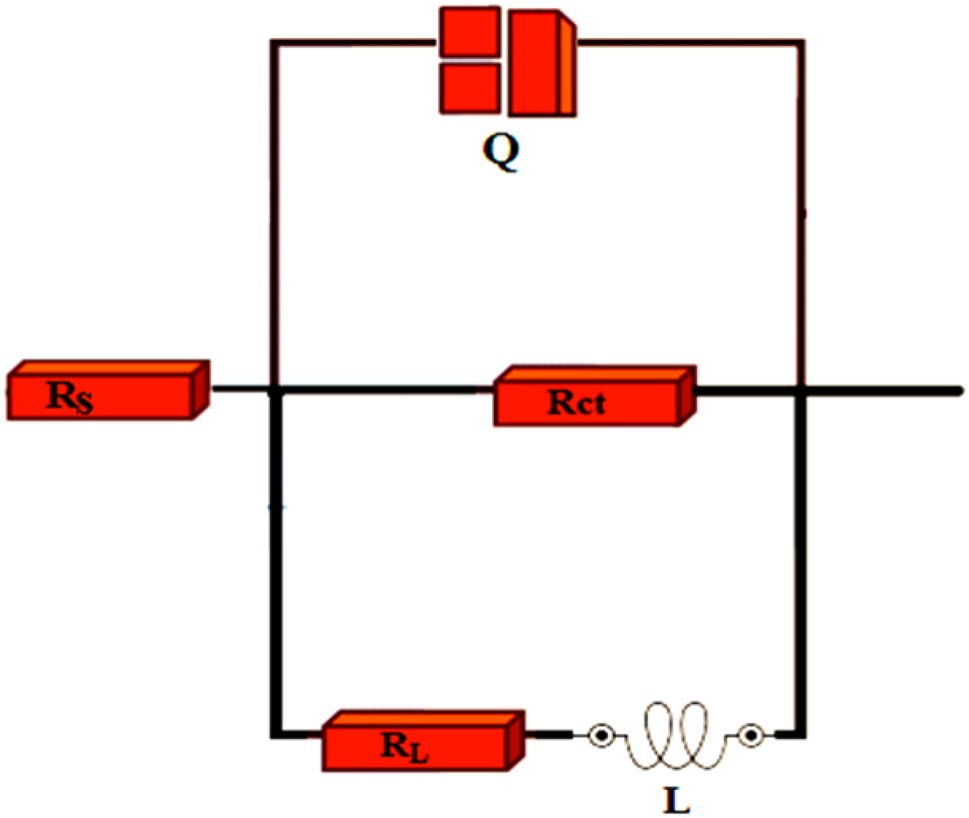
The equivalent circuit diagram to obtain the experimental data of EIS.

The corrosion mitigation process related to resistive and capacitive behaviors at the electrode/electrolyte interface was examined using the electrochemical impedance spectroscopy (EIS) investigations, conducted on the alloy of Al in 3.5% NaCl solution in the presence and absence of RA/Ag NPs. Figures 11-a and 11-b depict Nyquist, Bode and Bode phase plots, the single semicircle configuration of all Nyquist plots reveals no changes in the charge transfer mechanism of the corrosion process related to the alloy in solution without and with different concentrations of inhibitor^36,52^. The diameter of Nyquist plots increased along with increasing inhibitor concentration up to 200 ppm, because of the inhibitor adsorption enhancement on the active sites of metal surface, resulting in enhanced corrosion inhibitory behaviors^53–55^. Moreover, there was a depression in the Nyquist plots semicircle because the surface is rough and the metal electrode from the corrosion process is heterogeneous. Each relaxation process in the blank Bode-phase plots and the inhibitor-containing electrolyte can be associated with one semi-circle capacitive loop in the Nyquist plots within the frequency domain, indicating a time constant in the electrochemical system^56^. Fitting of the EIS experimental data took place with electrical equivalent circuit models (Fig. 12), containing a solution resistance (R_s_), an inductance (L), an inductive resistance (R_L_) series combination, along with charge transfer resistance (R_ct_) and a constant phase element (CPE)^36^. The obtained parameters through impedance estimations to explore the Al alloy’s corrosive function are given in Table 5, including phase shift (n), polarization resistance (R_p_), double layer capacitance (C_dl_), and inhibition performance (IE%)^57^. The polarization resistance (R_p_) can be calculated by below equation^36^:

**Table 5:** Corrosion parameters computed of Nyquist curves of Al alloy (100 mm^2^) in absent and presence of varies concentrations of RA/AgNPs.

C/ppm	CPE		R_ct_/kΩ cm^2^	C_dl_/mF cm^−2^	L/kH cm^2^	RL/kΩ cm^2^	Rp/kΩ cm^2^	IE%
	Y_0_ (μF cm^−2^)	n						
Blank	244	0.666	1.12	3218.71	0.66	1.50	0.64	–
50	95	0.764	2.56	410.28	1.86	2.81	1.33	51.87
100	27.4	0.823	4.22	106.11	2.20	2.98	1.74	63.21
150	27.3	0.822	6.48	66.26	5.50	9.50	3.85	83.37
200	10.9	0.858	9.50	2478	9.90	9.90	4.84	86.77
250	24.5	0.818	8.00	35.85	8.80	9.59	4.36	85.32

7 $${R}_{p}=\left(\frac{{R}_{ct}\times {R}_{L}}{{R}_{ct}+{R}_{L}}\right)$$

The inhibition efficiency can be obtained from polarization resistance (R_p_) according to Eq. (1). The results obtained by employing of this method confirm the increased R_ct_ values by increase inhibitor concentration up to 200 ppm, highlighting the protective film has been formed at the interface of metal and solution because the banner molecules are adsorbed on the alloy resulting block the corrosive ions accessibility, and mitigate the Al dissolution rate. The equation below is used to calculate the double layer (C_dl_) capacitance^58^:

8 $${C}_{dl}={\left({Q}_{CPE} \times {R}_{ct}^{1-n}\right)}^{1/n}$$

In which, Q, R_ct_ and n represent the constant phase element, the charge transfer resistance, and the phase shift concerning the surface inhomogeneity degree, respectively. The formula below is used to calculate the value of the Q, expressed by admittance Y_0_ and power n^59^:

9 $${Q}_{CPE}={Y}_{0}{(jw)}^{n}$$

In which *j,* ω, and n represent the imaginary number (*j*^2^ = − 1), the angular frequency in rad s^−1^, and the phase shift concerning the surface inhomogeneity degree, respectively. For *n* = 0, CPE shows a resistance, for *n* = 1, a capacitance and for *n* = − 1 an inductance. The dimension of Y_0_ will sn/Ω, and for capacitance (C) equal to s/Ω or F. Regardless of these differences, Y_0_ has more applications to depict the corroding system’s capacitance^59^.

The C_dl_ value significantly decreased due to the introduction of the NPs, water molecules replacement with the inhibitors adsorbed onto the surface of the metal, decreasing the local dielectric constant and increasing electrical double layer thickness^60^. As a result, the alloy surface is protected against corrosion by a better protective film. According to Table 5, adding the inhibitor could improve the efficiency of the inhibition, with the greatest values reported equal to 86.77% for the solution that contained 200 ppm RA/Ag NPs and provided the best surface coverage. The acceptable corrosion inhibitory performance of RA/Ag NPs has been reported for the Al alloy in 3.5% NaCl solution^61^. The inhibitor is capable of absorption on the surface of the metal, interaction with Al ions, and subsequent restriction of the aggressive agent accessibility for the active sites. The reported values of IE are consistent the values derived from ECN finding.

### Surface observations

**Figure 13:**
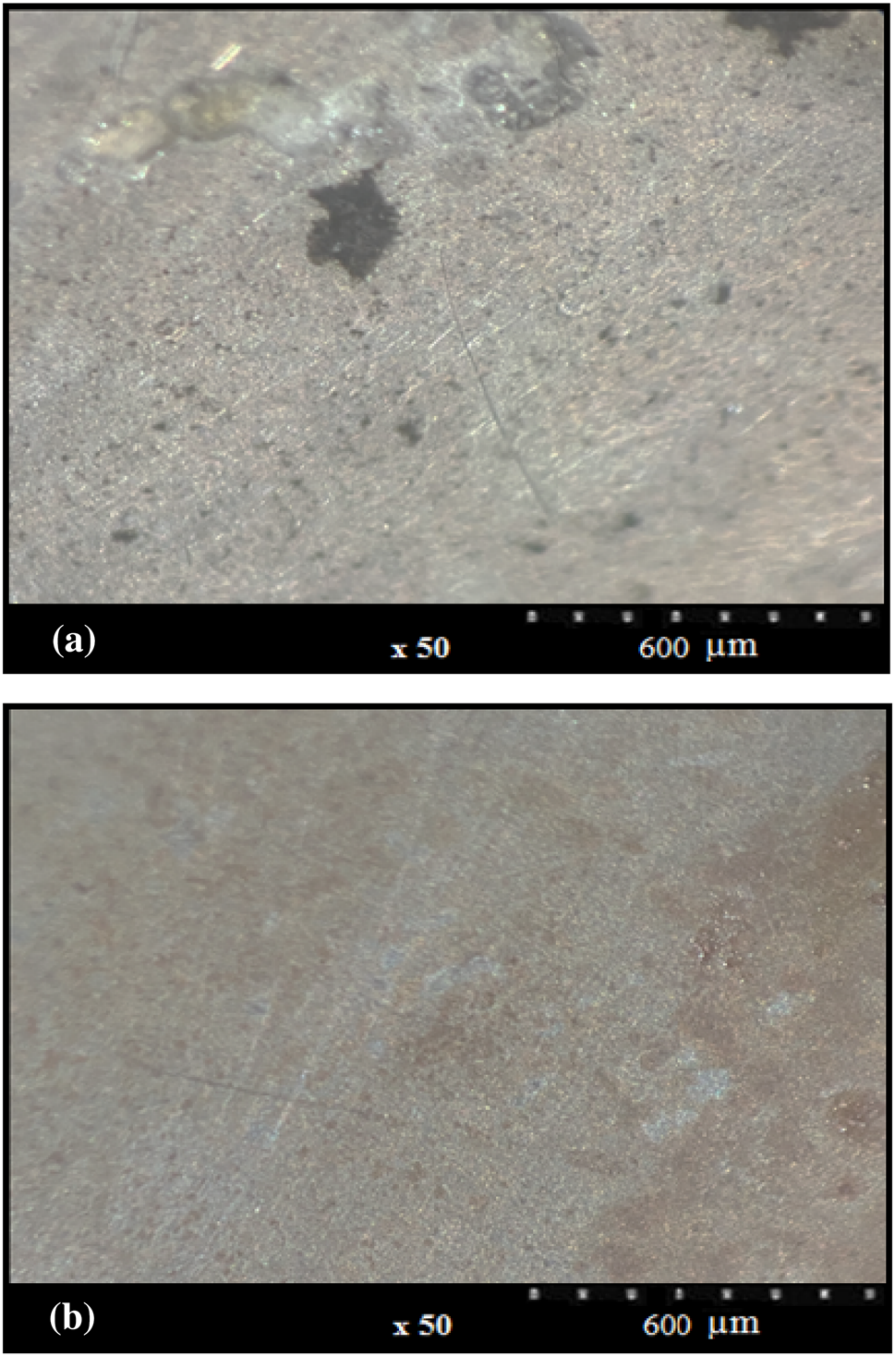
Optical microscopy images of Al alloy surface after 24 h immersion in 3.5% NaCl solution in the (**a**) absence, (**b**) presence of RA/AgNPs, respectively.

The use of the optical microscopy method aimed at achieving information on the Al surface morphology. Figure 13a,b indicate the relevant images of the alloy surface following 24 h of immersion in the solution of NaCl when the 200 ppm inhibitor concentration was absence and presence. Rigorous damage of the alloy surface is clearly in the absence of the inhibitor because of the metal surface degradation. On the other hand, the image by 200 ppm inhibitor showed smoother and less dissolution highlighting the passive film formation and the active sites obstruction. As confirmed by the surface specifications, the protective layer on the metal led to a significant reduction in the corrosion rate^62^.

## Conclusions

This research leads to the following:

1. Characterization methods have been shown that reducing agent aqueous extract of RA is suitable to synthesize RA/Ag NPs.
2. In the ECN signals arising from the As-Co in concentration up to 200 ppm, both the quantities of Q and standard deviations values of the transients decreased.
3. The As-Co made of Al alloys were more suitable for calculating the inhibiting effect of RA/Ag NPs compared to Sy-Co since the signal apparent maximum location in the SDPS plots of As-Co is actual and there is no requirement to look at the partial signal, while such a correlation is necessary for Sy-Co.
4. The ECN signals arising from As-Co presented the unidirectional values compare to Sy-Co due to recorded data are related to the small electrode and the large one is the substrate to consume the produced electrons.
5. The results obtained from the EIS measurements have been indicated that with increasing inhibitor concentration up to 200 ppm, the corrosion of 2030 aluminum alloy decreased.
6. The results derived from electrochemical current noise of As-Co and EIS measurements are in more accordance compare to Sy-Co.
7. The obtained results from ECN measurements and EIS displayed that RA/Ag NPs is appropriate inhibitor for AA 2030 aluminum alloy corrosion in a 3.5% NaCl solution.
8. The surface observations also validate the inhibition performance of the NPs.

## Data Availability

The datasets used and/or analysed during the current study available from the corresponding author on reasonable request.
